# Estimating Microbial Protein Synthesis in the Rumen—Can ‘Omics’ Methods Provide New Insights into a Long-Standing Question?

**DOI:** 10.3390/vetsci10120679

**Published:** 2023-11-27

**Authors:** Joana Lima, Winfred Ingabire, Rainer Roehe, Richard James Dewhurst

**Affiliations:** 1 SRUC Dairy Research and Innovation Centre, Barony Campus, Dumfries DG1 3NE, UK; joana.lima@sruc.ac.uk (J.L.); winfred.ingabire@sruc.ac.uk (W.I.); 2 SRUC, West Mains Road, Edinburgh EH9 3JG, UK; rainer.roehe@sruc.ac.uk

**Keywords:** microbial protein synthesis, rumen, microbiota, microbiome, urinary purine derivatives

## Abstract

**Simple Summary:**

Microbial protein is a valuable resource within the global food chain. It is produced by microbes inhabiting the rumen (i.e., the rumen microbiota), and provides at least half of the building blocks for the synthesis of milk and meat protein in ruminants. Our paper reviews experimental techniques previously used to estimate the quantity of microbial protein produced, and mathematical prediction models developed based on those previous studies. Earlier work involved direct sampling from the gut, whilst more recently the use of proxies such as urine purine derivatives to estimate microbial protein synthesis has been explored. Whilst the theory about microbial protein synthesis is well understood, predictions are not accurate. We show examples of newer lab techniques that identify relationships between the rumen microbiota and their genes (‘who is there?’ and ‘what are they doing?’) and host traits, e.g., methane emissions. We suggest that these techniques will enable better estimates and lead to more accurate predictions of microbial protein synthesis. We urge for a renewed programme of research using these techniques to describe and model protein degradation and synthesis in the rumen. These questions are fundamental to global food protein security and reduction in the environmental effects of ruminant livestock production.

**Abstract:**

Rumen microbial protein synthesis (MPS) provides at least half of the amino acids for the synthesis of milk and meat protein in ruminants. As such, it is fundamental to global food protein security. Estimating microbial protein is central to diet formulation, maximising nitrogen (N)-use efficiency and reducing N losses to the environment. Whilst factors influencing MPS are well established in vitro, techniques for in vivo estimates, including older techniques with cannulated animals and the more recent technique based on urinary purine derivative (UPD) excretion, are subject to large experimental errors. Consequently, models of MPS used in protein rationing are imprecise, resulting in wasted feed protein and unnecessary N losses to the environment. Newer ‘omics’ techniques are used to characterise microbial communities, their genes and resultant proteins and metabolites. An analysis of microbial communities and genes has recently been used successfully to model complex rumen-related traits, including feed conversion efficiency and methane emissions. Since microbial proteins are more directly related to microbial genes, we expect a strong relationship between rumen metataxonomics/metagenomics and MPS. The main aims of this review are to gauge the understanding of factors affecting MPS, including the use of the UPD technique, and explore whether omics-focused studies could improve the predictability of MPS, with a focus on beef cattle.

## 1. Introduction

Food production systems are under increasing pressure to meet global food demand due to the growing human population and food preference changes associated with increasing life quality standards [1,2]. Ruminants are particularly valuable because of their ability to utilise human indigestible feeds, providing high-quality protein-rich foods for human consumption, such as meat and milk, without necessarily competing for resources.

Although ruminants do not digest the complex polysaccharides in their feed, they live in a symbiotic relationship with their rumen microbiota, harbouring a complex consortium of microbial communities that feed on these substrates, and ferment them into volatile fatty acids, microbial protein, and vitamins that the host will absorb and use for their own maintenance and growth, and are therefore closely related to animal productivity.

Microbial protein provides at least half of the amino acids absorbed by ruminants under most feeding situations and as such represents a major challenge and opportunity for providing high-quality animal proteins in human diets [3]. Virtanen et al. [4] fed dairy cows with urea and paper to demonstrate the potential of ruminants to synthesise microbial protein from non-protein and fibrous materials, which is particularly important in developing countries with large deficits of protein foods.

The limited supply and carbon footprints of protein feeds for ruminants, such as soybean and palm kernel meals, is also of concern. Protein-rich feeds or protein additives are expensive for farmers. However, protein that escapes fermentation by the rumen microbiota and is not used by the host (together with endogenous protein) contribute to elevated urinary nitrogen (N) losses, leading to decreased profitability. In addition, urinary N losses can increase nitrate leaching to groundwater and emissions of nitrous oxide, a potent greenhouse gas [5], negatively impacting the environment.

Considering the close association between MPS and the ruminants’ productivity, profitability, and environmental impact, the accurate estimation of microbial protein synthesis (MPS) is necessary, not only due to its large contribution to global food protein security, but also to optimise protein supplementation to ruminants and minimise N losses to the environment. In particular, omics-focused investigations of MPS and the influence of rumen microbiota patterns on MPS are extremely relevant, because they can provide a holistic overview of the interactions and further improve MPS prediction accuracies.

Several previous authors have focused on predicting MPS from different factors (e.g., dietary energy, dietary digestible crude protein). These are reviewed here and discussed in the context of previously proposed prediction equations. In addition, we discuss methodological problems associated with MPS prediction in cattle. We also analysed 34 published studies that focused on the prediction of MPS based on urinary purine derivative (UPD) measurements. These studies included work on *Bos taurus* and *Bos indicus*, which were selected from the Web of Science platform using ‘purine derivatives’ and ‘beef cattle’ search words. The main objective of this systematic review is to understand whether omics-centred studies could improve the understanding of factors affecting MPS, and thereby improve on extant MPS predictions.

## 2. Energy Supply Limiting Microbial Protein Synthesis

Several factors affect MPS and have been reviewed by Pathak et al. [6], including dry matter intake, passage rates, and rumen environmental conditions. The most important factors identified are the energy and protein supplied to the rumen microbiota.

The theoretical basis for variation in MPS is well established, including the effects of the increasing supply of chemical energy in the form of adenosine triphosphate (ATP), as represented by dietary fermentable organic matter (OM), and the effects of the microbial growth rate on the relative proportions of ATP used to support microbial maintenance and growth functions (see references and discussion in [7]).

In addition to the energy provided in the diet, the timing of the release of this energy to be used by the rumen microbiota influences the MPS efficiency. Researchers looking into short-term fluctuations of the rumen microbiota have identified four phases after the feed enters the rumen, including a displacement of the epiphytic microbiota; primary colonization, characterized by growth of microbes that ferment easily digested carbohydrates (with the production of ATP); stationary phase; and then a second colonization phase, with a growth of cellulose- and hemicellulose-fermenting microbes (with the production of ATP) [8,9,10,11,12,13]. Although the rapid fermentation of non-fibre carbohydrates provides ATP that can be used for microbial growth, the associated drop in pH negatively affects fibre-degrading microbes, which may lead to decreased MPS. The formulation of diets considering the two feed colonization events may maximise MPS by allowing for continuous ATP production in the rumen [14].

The term Y_ATP_ is used to describe the yield of microbial material per mole of ATP; Pirt [15] developed equations to describe the effects of the microbial growth rate on Y_ATP_. Whilst additional fermentable OM supply from well-balanced diets may increase MPS, increasing the proportion of concentrates added to low-quality forage-based diets has been shown to lead to decreased MPS. This has been associated with decreased pH from the rapid production of volatile fatty acids negatively affecting bacteria, due to the redirection of their energy towards coping with low pH rather than to growth and/or fermentation [6]. On the other hand, the authors also mention that low pH negatively affects protozoa, and that rumen defaunation is associated with improved MPS [6]. This highlights the need for holistic studies to explore the associations between different taxa and their functional potentials affecting MPS.

Higher passage rates from the rumen have been associated with higher microbial growth rates, which may explain the higher efficiency of MPS in higher producing animals with higher rumen passage rates. This has been demonstrated using rumen in vitro systems [16].

A series of empirical models have been developed over the last five decades to predict MPS from energy supplied to rumen microbes, based on estimates from studies with post-rumen-cannulated animals. The initial models reflected the effect of the energy supply on MPS, whilst more recent models are more complex, also considering the effects of diet types, the level of feeding (which affects rumen passage rates), and the fermentable energy available to rumen microbes [17,18]. Many of the early studies were conducted using sheep. Very few such studies have been conducted in recent years; thus, the testing of models has been limited. Table 1 provides a summary of some of the equations used to predict MPS.

MPS/FME (g/MJ) = 7 + 6 × (1 − e^−0.35 × Level of feeding^) (1)

FME = ME − ME_fat_ − ME_fermentation acids_ (2)

TDN = digestible CP + digestible CF + digestible NFE + 2.25 × digestible EE (3)

FFTDN = digestible CP + digestible CF + digestible NFE (4)

For dairy cattle in the UK, the Feed into Milk project [22] developed a more complex model to predict MPS using estimates of the production and efficiency of the utilisation of ATP by rumen microbes. This approach partly explains the higher MPS per unit of fermented energy observed in more productive animals, such as high-yielding dairy cows. However, the Feed into Milk publication did not provide a direct evaluation of models; justification for the model was based on predictions matching the mean and range of values in a comprehensive review of MPS by Archimède et al. [23], and the fact that the whole Feed into Milk model provided a good fit to results from production studies with dairy cows. We have reached the limit of advances that can be obtained through further measurements and modelling based on estimates of MPS using rumen-cannulated animals.

There are many theoretical reasons why these models fail to describe much of the variation in MPS, such as differences in rumen turnover rates, the maintenance energy requirements of individual microbial species [16,24], and the extent and cost of microbial cross-feeding, for example, the effects of the protozoal predation of bacteria—as illustrated by the large increases in the efficiency of MPS (per unit fermented OM) when rumens are defaunated [25]. As discussed later, the other major reason for the low proportion of variation explained by these models is the large experimental errors associated with the techniques used.

## 3. Protein Supply Limiting Microbial Protein Synthesis

Rumen-degraded nitrogen (protein; RDN/RDP) includes true protein N (including peptides) and non-protein N (nucleic acids, amino acids, and ammonia). Peptides, amino acids, and ammonia are the building blocks used by rumen microbes to synthesise microbial protein. Whilst rumen microbes can grow without pre-formed amino acids, some are stimulated by them [26]. Therefore, RDP/RDN can constrain MPS (as well as energy, as explained in Section 2).

Previous studies using cannulated animals have shown that dietary crude protein (CP) content and digestibility influence microbial growth and MPS [24,25,26], and that this is particularly important to meet the metabolisable protein requirements of high-yielding dairy cows [27].

The most recent review of MPS from beef cattle was conducted by Galyean and Tedeschi [21] using studies reported in the Journal of Animal Science. This review found lower estimates of MPS than previous models for beef cattle used in the US. As the authors noted, this is most probably related to the low RDN supply in many of the diets based on maize and mature hays that were used in the studies under review; they commented that for 202 out of 285 treatment means in their analysis, MPS was most likely limited by protein—rather than energy—supply and so was calculated as RDP intake multiplied by 0.85.

The French rationing system [28] adopted an elegant approach to address this constraint in practical rationing by defining metabolisable protein (PDI; protein digestible dans l’intestin) for situations in which MPS is constrained by energy supply (PDIE) or by RDN supply (PDIN). The rationing software uses whichever is lower (most constrained), PDIE or PDIN.

## 4. Methodological Problems

The main limitation for further meaningful improvements of these prediction models is the techniques available to estimate MPS in vivo. Although work with intestinally cannulated animals has provided interesting information, for example on the influence of dietary CP content on microbial growth (as mentioned in Section 3), this type of experimental setup is difficult, invasive, and subject to considerable experimental errors. Challenges related to the use of cannulated animals include the difficulty in obtaining representative samples of digesta and microbes from the digestive tract, estimating the flow of digesta at the abomasum or duodenum, and in distinguishing microbial protein from undegraded dietary protein or endogenous proteins in digesta, as discussed by Dewhurst et al. [7]. A wide range of markers were used to identify microbial protein in digesta sampled from the abomasum or duodenum, both internal (e.g., purine bases and diaminopimelic acid, which are mostly confined to microbial material) and external (e.g., the incorporation of ^15^N or ^35^S). Offer et al. [29] used the amino acid profile of feeds, microbes, and endogenous (animal) material to estimate relative proportions of amino acids from these three sources in digesta. McAllan and Smith [30] developed the use of DNA and RNA in samples of digesta from cattle as markers for microbial protein; this was further developed by Zinn and Owens [31] using a simpler method to analyse purine bases as microbial markers. Furthermore, previous authors have shown that MPS estimated based on purine N from duodenum digesta was not significantly different to that estimated from urinary purine derivatives’ excretion [32].

Over the last two decades, extensive research has been conducted using the excretion of purine derivatives in urine as a biomarker for rumen MPS based on studies, e.g., by Chen et al. [33,34] exploring relationships between absorbed (or infused) purines and the excretion of purine derivatives in urine. The underlying principle is that the purine bases (adenine and guanine) in nucleic acids are produced by the rumen microbiota, absorbed in the small intestine, and metabolised into purine derivatives (mostly allantoin and uric acid), which are mostly excreted in urine.

One of the main challenges to estimate MPS from purine derivatives in urine is differences in purine metabolism in different species of animals and animal production systems. In cattle, practically all absorbed purines are converted by xanthine oxidase into uric acid, which enters the liver and is therefore not available to be used by the animal. In sheep, purines are not converted, and therefore can enter the liver and be available to be used by the animal, and only the purines that are not incorporated into tissue nucleic acids will contribute to purine derivatives in urine [34]. In addition, in dairy animals, some uric acid and allantoin (both purine derivatives) will be excreted in the milk [35].

Whilst the urinary purine derivative technique provides a non-invasive approach, it is still limited by the lack of information about the composition (purine content) of rumen microbes. Nonetheless, it has become much more prevalent than techniques using intestinal cannulation.

## 5. Analysis of Factors Affecting Urinary Purine Derivative Excretion

UPD excretion has been proposed as an alternative and less invasive approach to the prediction of MPS. However, these predictions are only valid if biochemical mechanisms associated with UPD are common between varying animals. Therefore, we explored how factors such as diet, feed intake, and animal weight are associated with UPD in two different breed types, *Bos taurus* and *Bos indicus*.

### 5.1. Methods

We reviewed 34 published studies [36,37,38,39,40,41,42,43,44,45,46,47,48,49,50,51,52,53,54,55,56,57,58,59,60,61,62,63,64,65,66,67,68,69] that were obtained by searching within the Web of Science platform using ‘purine derivatives’ and ‘beef cattle’ as keywords for the document search. We only included studies that reported results for UPD excretion alongside recording feed dry matter (DM) intake, animal weight, and dietary components (CP and neutral detergent fibre (NDF) on a DM basis). The data included means from 138 groups of animals, of which 69 were *Bos taurus*, 51 were *Bos indicus,* and 9 were Crossbreds (crossbreeding between *Bos indicus* and *Bos taurus*).

Since UPD was not normally distributed over all 138 groups (Shapiro test, *p* = 0.00002378), and the data were not balanced for breeds, we performed a Kruskal–Wallis rank sum test to compare the mean UPD of species. In addition, we performed pairwise Kruskal–Wallis rank sum comparisons post hoc to evaluate species differences. *p*-values were adjusted based on the false discovery rate (FDR, Benjamini and Hochberg, 1995). Crossbred animals were removed from further analyses due to the small number of records (n = 9). Pearson correlations were estimated within species between CP (dry matter (DM) basis), NDF, dry matter intake (DMI), DMI/weight, and CP/NDF. Correlations’ significance was calculated, and *p*-values were corrected using the FDR. To evaluate the association of UPD with CP, NDF, DMI, and DMI/weight within species (*Bos indicus* and *Bos taurus*), linear models were applied within species. The dependent variable in both models was UPD, which was log-transformed using the natural logarithm to approach a normal distribution of its values. The explanatory variables included in the models were CP/NDF (to account for the compositionality of the diet composition data), DMI, DMI/weight, and the interaction between DMI and DMI/weight. Within *Bos taurus*, the interaction was not significant, and was therefore removed from the model.

All statistical analyses were performed using R software [70] and package openxlsx [71].

### 5.2. Results

Pearson correlations (Table 2) revealed low to moderate associations of UPD with CP (0.36 and 0.55 for *Bos taurus* and *Bos indicus*, respectively), and NDF (−0.28 and −0.44 for *Bos taurus* and *Bos indicus*, respectively). The correlation between UDP and DMI was significant and moderate within *Bos taurus*, but not significant within *Bos indicus*. UPD was not correlated with DMI/weight (R = 0.03 and –0.04 in *Bos taurus* and *Bos indicus*, respectively). In addition, moderate correlations were observed between CP and NDF dietary content (−0.61 and –0.40 in *Bos indicus* and *Bos taurus*, respectively), which was expected because these variables are not statistically independent (both are components of the same unit).

NDF was moderately associated with DMI/weight when the analysis was performed within *Bos indicus* (−0.43) but showed a non-significant correlation within *Bos taurus* (0.06).

The linear models revealed a moderate association of UPD with CP, NDF, DMI, and DMI/weight. Within *Bos taurus*, CP/NDF, DMI, and DMI/weight explained 34% of the variance in UPD, whereas within *Bos indicus*, these variables plus the interaction between DMI and DMI/weight explained 45% of the variance in UPD (Table 3).

The first interesting finding from our analysis is the difference in relationships between *Bos taurus* and *Bos indicus* cattle in agreement with Gandra et al. These authors reported higher UPD excretion per unit DM intake for *Bos indicus* cattle (7.29 versus 5.98 mmol UPD/kg DMI for *Bos indicus* and *Bos taurus* cattle, respectively). This may reflect differences in the purine metabolism of the different breeds, differences in rumen function, or both. There are significant differences in rumen fermentation rates and digesta kinetics between these breeds; these also interact with differences in diet composition, particularly dietary protein levels, reflecting differences in urea recycling to the rumen (see the review by Hegarty [72]). Although Prates et al. [73] found no differences in purine metabolism between Nellore (*Bos indicus*) and Holstein (*Bos taurus*) heifers, earlier studies did suggest differences [74]. Accordingly, we analysed results from the two species separately.

Our analysis suggests that diet composition, represented by the protein/NDF ratio, explained more variation in UPD excretion than DM intake. The increased MPS in response to dietary protein may partly explain why growth rate responses in cattle offered more protein than needed to meet metabolisable protein requirements. A recent study of the microbial community and rumen metabolome of goats offered diets with differing protein content showed complex effects that should be incorporated into future models of MPS [75]. The effect of protein on MPS is much more complicated than simply being a supply of amino acids or ammonia. We were using DM intake (g/day or as a % of body weight) as a proxy for rumen passage rates and found no evidence for effects on UPD excretion. This may reflect the narrow range of feeding levels encountered with beef cattle, compared with, for example, high-yielding dairy cows.

As with results from studies with cannulated cattle, there remains a substantial proportion of variation in MPS that is not explained, further highlighting the need for new approaches (next section). There are several sources of experimental error in the use of the UPD technique to estimate MPS, including the duration of sample collection (including use of ‘spot sampling’ in some studies), as well as methods for sample preservation and analysis. UPD were significantly different between groups (*p* < 0.05), with averages ± standard deviations of 126.0 ± 43.7, 83.2 ± 37.0 and 175.4 ± 57.6 within *Bos indicus, Bos taurus,* and their crossbred animals, respectively. Post hoc pairwise comparisons confirmed that all groups differed significantly from each other.

## 6. Microbiome Approaches to Predict Rumen-Related Traits

At around the same time that rumen biologists such as McAllan and Smith [30] were developing analytical techniques to measure nucleic acids or their component bases in digesta, molecular biologists were developing a series of ‘omics’ techniques to isolate nucleic acids from samples, sequence the DNA or RNA, and quantify proportions of different microbial taxa (metataxonomics), genes (metagenomics), or gene expression (metatranscriptomics).

These omics techniques provide an extensive description of the rumen microbial community and its activity and have been extremely useful in providing rapid predictions of complex rumen-related traits, such as methane emissions and feed conversion efficiency, that are difficult to measure using traditional experimental approaches and are not amenable to use with large numbers of animals in commercial settings. Table 4 and Table 5 provide recent examples of this approach for beef cattle and dairy cattle, respectively.

We recommend research to use these omics approaches to estimated MPS, which we expect to be related strongly to microbial taxa and genes and their metabolism in the rumen. The link between metagenomes or metatranscriptomes and MPS is expected to be more direct, since the relationships of rumen-related growth traits depend on MPS that in turn is based on actions of microbial proteins, such as microbial enzymes. In particular, microbial KEGG genes are highly informative because their functions in, e.g., metabolic pathways are known and could therefore be expected to provide a better estimation of MGS. Recently, Martinez-Alvaro [86] found that diverting substrates in the rumen for MPS will reduce the metabolism of the highly potent greenhouse gas methane in the rumen, which would be an advantage for animal growth with a simultaneous reduction in the environmental impact of ruminant livestock production. Earlier studies showed that the concentrations of DNA and RNA in microbial material are affected by microbial species and growth rate (Arambel et al. [90]; Bates et al. [91]). The analysis of genes and gene expression in the rumen could help with the modelling of rumen MPS, as developed in other microbial systems by Muscarella et al. [92]. Muscarella explored the relationships between metagenomics, metataxonomics, and nutrient supply on bacterial growth efficiency, and the partitioning of assimilated carbon between biomass production and maintenance functions which result in the release of carbon dioxide. It is well recognised that there are large differences in the maintenance energy expenditure of different rumen bacteria, relationships with maximum growth rates of different taxa and effects on the structure of microbial communities (e.g., Russell and Baldwin [93] and Russell and Cook [94]); research is needed to understand how this relates to the different genes and metabolic functions. Some of the variation in MPS may relate to genetic differences between animals, effects that have not been explored because of the technical challenges of estimating MPS in studies with large numbers of animals. Omics techniques, in this case known as host genomics, in combination with rumen metataxonomics and metagenomics, can help explore these effects, in a comparable way to studies of methane emissions included in Table 5.

The challenge remains that it is difficult to separate out microbial protein from feed protein that has not been broken down in the rumen (undegradable dietary protein) and endogenous (animal) proteins, such as enzymes and sloughed cells that are present in digesta. In contrast to the examples of methane emissions and feed conversion efficiency, there is no ‘Gold Standard’ method for MPS that could be used to calibrate an ‘omics’ approach.

Recent advances in metaproteomics will allow us to take a similar approach to the laborious amino acid profiling technique of Offer et al. [29], using peptide sequences to distinguish feed, microbial, and endogenous proteins in digesta. As we gather more information about the amino acid sequences of more proteins, it will become possible to provide a detailed description of complex mixtures of proteins, such as is found in digesta samples.

## 7. Conclusions

Microbial protein is a particularly valuable resource within the global protein food chain. Research to quantify this valuable resource was hampered by the difficulties of working with cannulated animals, as well as difficult marker techniques. Our review suggests that omics-focused studies are essential to understand the dynamics between the dietary composition, rumen microbiome profiles, and MPS, particularly to improve the MPS prediction accuracy and profitability of animal production systems. We urge for there to be a renewed programme of research using metataxonomics, metagenomics, metatranscriptomics, and metaproteomics to describe and model protein degradation and synthesis in the rumen.

## Figures and Tables

**Table 1:** Equations to predict rumen microbial protein synthesis (MPS; g/day) based on estimates of energy supplied to rumen microbes.

Reference	Predicted MPS
ARC (1980) [19]	7.81 × ME intake (MJ/day)
ARC (1984)—Well balanced mixed diets [20]	8.4 × ME intake (MJ/day)
ARC (1984)—Solely grass silage [20]	6.25 × ME intake (MJ/day)
ARC (1984)—Grass silage & concentrates [20]	8.75 × ME intake (MJ/day)
AFRC (1992) equation ^ǂ^—Applied to maintenance level of intake [17]	8.8 × FME intake (MJ/day)
AFRC (1992) equation ^ǂ^—Applied to 2× maintenance level of intake [17]	10.0 × FME intake (MJ/day)
AFRC (1992) equation ^ǂ^—Applied to 3× maintenance level of intake [17]	10.9 × FME intake (MJ/day)
AFRC (1992) equation ^ǂ^—Applied to 4× maintenance level of intake [17]	11.5 × FME intake (MJ/day)
NASEM (2016): beef cattle—Based on Galyean and Tedeschi (2014) (for dietary EE < 3.9% DM) [21]	42.73 + 0.087 × TDN intake (g/day)
NASEM (2016): beef cattle—Based on Galyean and Tedeschi (2014) (for dietary EE > 3:9% DM) [21]	53.33 + 0.096 × FFTDN intake (g/day)

ME, FME, TDN, FFTDN, EE, CP, CF, NFE, and DM refer to metabolisable energy, fermentable ME, total digestible nutrients, fat-free TDN, ether extract, crude protein, crude fibre, nitrogen-free extract, and dry matter, respectively. ARC, AFRC, and NASEM refer to Agricultural Research Council, Agricultural and Food Research Council, and National Academies of Sciences, Engineering, and Medicine, respectively. ^ǂ^ shown in Equations (1)–(4).

**Table 2:** Pearson correlations of urinary purine derivatives with dietary components (protein and fibre), and performance traits (dry matter intake and dry matter intake per weight) extracted from previous studies on beef cattle (*Bos taurus* and *Bos indicus*).

	UPD	CP	NDF	DMI	DMI/Weight	CP/NDF
**UPD**		0.55 *	−0.44 *	0.16	−0.04	0.56 *
**CP**	0.36 *		−0.61 *	−0.17	0.03	0.81 *
**NDF**	−0.28 *	−0.40 *		−0.05	−0.43 *	−0.91 *
**DMI**	0.38 *	0.10	0.06		0.29	−0.18
**DMI/weight**	0.03	0.26	−0.10	0.57 *		0.22
**CP/NDF**	0.31 *	0.71 *	−0.86 *	−0.03	0.20	

UPD, CP, NDF, and DMI refer to urine purine derivatives, crude protein, neutral detergent fibre and dry matter intake, respectively. Cells coloured in yellow and blue correspond to correlations calculated within *Bos indicus* and *Bos taurus* groups, respectively. The * refers to significant correlations (*p* < 0.05). For this analysis, studies [36,37,38,39,41,42,43,44,46,47,48,49,50,51,52,53,54,55,56,57,58,59,60,61,62,63,64,65,66,67,68,69] were included.

**Table 3:** Results from linear regressions of urinary purine derivative excretion on crude protein, neutral detergent fibre, and dry matter intake within species (*Bos taurus* and *Bos indicus*).

Species	*Bos taurus*	*Bos indicus*
Explained variance (R^2^)	34%	45%
Explanatory variables	CP/NDF + DMI + DMI/weight	CP/NDF + DMI + DMI/weight + DMIDMI/weight
*p*-value	0.00000154	0.00000172
**Regression coefficients**		
Intercept	3.86	3.15
CP/NDF	1.20	1.11
DMI	0.10	0.22
DMI/weight	−32.59	26.88
DMI*DMI/weight	NA	−5.15

Only factors with significant regression coefficients at *p* < 0.05 were fitted in the linear model (NA = non-applicable).

**Table 4:** Use of omics techniques to estimate rumen-related traits in beef cattle.

Reference	Trait (Units)	Range	Omics Technique	% of Variation Explained
[76]	RFI (kg/d)	−1.38 ± 0.14 to 1.40 ± 0.12	PGR-DGGE	Rumen bacterial profiles clustered separately for highly and lowly efficient animals
			Metabolomics	Animals with varying RFI had different rumen VFA concentrations
[77]	CH_4_ (g/kg DMI)	13.43 to 25.26	Metataxonomics	High emitters and low emitters differed due to 9 bacterial phyla, 5 bacterial genera, 1 archaeal phylum, and 2 archaeal genera
			Metagenomics	88% (20 microbial genes + diet)
[78]	CH_4_ (g/kg DMI)	14.4 to 31.4	Metagenomics	81% (20 microbial genes)
	FCR (DMI kg/ADG kg)	6.1 to 10.4	Metagenomics	86% (49 microbial genes)
[79]	CH_4_ (g/kg DMI)	20.89 ± 0.75	Metagenomics	62% (37 microbial genes + diet + breed)
			Metataxonomics	50% (56 microbial genera + diet + breed)
			Metagenomics + metataxonomics	42% (37 microbiome factors including microbial genes, microbial genera, diversity indices, and A:B ratio)
[80]	CH_4_ (g/kg DMI)	7.64 to 30.37	Metagenomics	Identification of 3 clusters of 237, 91, and 41 genes divergent between high and low methane emitters. Out of 91, 36 genes were in the methane metabolism pathway
[81]	Feed efficiency (FCR, RFI)		Metagenomics	379 microbial genera out of 1058 significantly diverged between animal groups (high efficiency vs. low efficiency)
	FCR	5.11 to 11.91	Metagenomics	39% (8 microbial genes)
			Metataxonomics	60% (45 microbial genes)
	RFI	−1.52 to 1.58	Metagenomics	40% (8 microbial genes)
			Metataxonomics	52% (85 microbial genera)
[82]	FCR	5.16 to 11.91	Metagenomics	63% (20 microbial genes)
	ADG	0.89 to 2.12		65% (14 microbial genes)
	DFI	8.52 to 18.76		66% (17 microbial genes)
	RFI	−1.76 to 4.16		73% (18 microbial genes)
[83]	CH_4_ (g/kg DMI)	17.56 with CV of 0.13	Metagenomics	57% (5 microbiome features representing a methanogenic cluster) 50% and 38% (sets of 5 microbiome features representing groups of fibre-degrading microbes)
[84]	CH_4_ (g/kg DMI)	Mean = 13.47	Genomics and metagenomics	166 microbiome features (including microbial genes, genera, and metagenome-assembled and uncultured genomes) had genetic correlations between |0.59| and |0.93| with methane emissions
[85]	Fatty acid profiles + CH_4_	NA	Genomics and metagenomics	27.6% (1002 microbial genes) of the functional core microbiome (3631 microbial genes) has heritability from 0.20 to 0.58 372 heritable microbial genes were involved in microbial metabolic pathways associated with unsaturated fatty acids with health benefits for humans (N3), hypercholesterolemic saturated fatty acids (CLA), or both. Microbiome-driven breeding of animals for improved N3 and CLA would include 31 microbial genes. A correlated response to selection on methane emissions ranged from reductions of 4% to 9% per generation (depending on intensity of selection)

CH_4_, FCR, ADG, DMI, DFI, RFI, VFA, A:B, and NA refer to methane emissions, feed conversion ratio, average daily weight gain, dry matter intake, daily feed intake, residual feed intake, volatile fatty acids, Archaea:Bacteria, and non-applicable, respectively. PCR-DGGE refers to polymerase chain reaction-denaturating gradient gel electrophoresis.

**Table 5:** Use of omics techniques to estimate rumen-related traits in dairy cattle.

Reference	Trait (Units)	Range	Omics Technique	% of Variation Explained
[86]	CH_4_ (g/d)	282 to 408	Metataxonomics	26 OTUs divergent between high and low emitters
[87]	Acetone (AC) and β-hydroxybutyric acid (BHB)—susceptibility to ketosis	Mean_AC_ = 0.57 with CV 1.24; Mean_BHB_ = 0.89 with CV 0.59	Metataxonomics	15%
[88]	Milk protein (%)	3.33 ± 0.35	Metataxonomics	8%
	Milk fat (%)	4.00 ± 0.77	Metataxonomics	8%
	Fatty acids in milk (C15:0) (%)	1.11 ± 0.24	Metataxonomics	42%
	Fatty acids in milk (C18:3 n − 3) (%)	0.53 ± 0.09	Metataxonomics	31%
[89]	Milk protein yield (kg/d)	Mean_LOW_ = 0.70 and mean_HIGH_ = 1.23	Metataxonomics	18%
			Metagenomics	22%
			Metabolomics	30%

CH_4_, OTU, NA, and CV refer to methane, operational taxonomic units, not available, and coefficient of variation, respectively.

## Data Availability

Not applicable.
